# Outcomes and Prognostic Analysis of Therapeutic Bronchoscopy in Non–Small Cell Lung Cancer (NSCLC) Patients With Respiratory Failure Secondary to Malignant Central Airway Obstruction (MCAO)

**DOI:** 10.1155/pm/4702786

**Published:** 2026-07-20

**Authors:** Xiuyan Liu, Yukun Tao, Jing Li, Ming Ding

**Affiliations:** ^1^ Department of Respiratory and Critical Care Medicine, Nanjing Central Hospital, Nanjing, Jiangsu, China; ^2^ Department of Anesthesiology, Zhongda Hospital, Southeast University, Nanjing, China, seu.edu.bd; ^3^ Department of Respiratory and Critical Care Medicine, Southeast University Zhongda Hospital, Nanjing, Jiangsu, China

**Keywords:** central airway obstruction, non–small cell lung cancer, respiratory failure, therapeutic bronchoscopy

## Abstract

**Objective:**

The purpose of this study was to investigate the value of therapeutic bronchoscopy in malignant central airway obstruction (MCAO) patients secondary to non–small cell lung cancer (NSCLC), especially in those experiencing respiratory failure (RF).

**Methods:**

This retrospective cohort study was conducted on 101 cases of NSCLC‐related MCAO patients who received therapeutic bronchoscopy from January 2018 to January 2025. Patients were classified into the RF group (*n* = 39) and the non‐RF group (*n* = 62) based on the presence of RF (PaO_2_/FiO_2_ ≤ 300) resulting from MCAO.

**Results:**

Therapeutic bronchoscopy achieved technical success in 93.1% of 101 patients, with no significant differences in technical success (*p* = 0.521), complication rates (*p* = 0.147), and short‐term or intermediate‐term efficacy between RF and non‐RF groups. Kaplan–Meier curves indicated a median survival of 16.98 months posttherapeutic bronchoscopy, with univariate analysis revealing statistically significant differences between the RF and non‐RF groups (10.46 months vs. 18.62 months, log − rank *χ* ^2^ = 4.494, *p* = 0.034). Multivariate Cox survival regression indicated that ECOG PS of 3–4 points (HR = 3.437, 95% CI: 1.751–6.746, *p* < 0.001), serum lactate dehydrogenase (LDH) ≥ 250 U/L (HR = 2.538, 95% CI: 1.328–4.852, *p* = 0.005), and lack of systemic antitumor treatment (HR = 3.274, 95% CI: 1.481–7.235, *p* = 0.003) are risk factors affecting the 2‐year survival of NSCLC‐related MCAO patients. Notably, the presence of secondary RF, the location, and the degree of airway stenosis in patients before surgery were not associated with the prognosis (*p* > 0.05).

**Conclusion:**

Therapeutic bronchoscopy is safe and effective in NSCLC patients with MCAO. This intervention should be performed promptly when airway narrowing causes RF. The ECOG PS, serum LDH level, and systemic antitumor treatment might be independent factors affecting the prognosis of MCAO patients.

## 1. Introduction

Malignant central airway obstruction (MCAO) is characterized by any malignant condition that severely impairs the patency of the trachea, left and right main bronchus, and the right intermediate bronchus with a stenosis degree of ≥ 50% [1, 2]. Approximately 30% of patients with lung cancer will progress to MCAO [3, 4], leading to ventilation challenges and comorbidities like dyspnea, hemoptysis, and respiratory failure (RF). Although surgical resection of the tumor‐involved airway followed by airway reconstruction represents the optimal treatment option, 88% of patients with MCAO are diagnosed at advanced stages of cancer, with no surgical indications or an inability to tolerate surgical therapy [5]. Meanwhile, chemotherapy and radiotherapy are generally insufficient for the rapid alleviation of acute symptoms associated with severe airway stenosis. Based on the situation, therapeutic bronchoscopy is widely acknowledged in integrated therapy for MCAO patients due to its immediate relief of airway obstruction and symptoms [6–8].

There is great heterogeneity in the primary diseases of MCAO, with common pathologic types including lung cancer (54.08%), esophageal cancer (22.45%), and thyroid cancer (3.06%) [9]. Although non–small cell lung cancer (NSCLC) occupies the highest incidence among primary lung tumors, few studies are available on the outcomes and prognosis of therapeutic bronchoscopy in MCAO patients secondary to NSCLC. In addition, as the most severe comorbidity of MCAO, RF is also a relative contraindication for bronchoscopy operations. However, the effectiveness of conventional treatment is limited when MCAO‐related RF occurs and may even delay interventional procedures, leading to deterioration. Although bronchoscopic relief of airway obstruction can reverse RF in selected patients [10–12], no reliable evidence confirms whether therapeutic bronchoscopy provides long‐term survival benefits for MCAO‐induced RF.

Therefore, the primary objective of this study is to investigate the efficacy of therapeutic bronchoscopy in patients with NSCLC‐related MCAO, focusing on technical success, safety, and prognostic outcomes. It also evaluates the benefit of RF patients secondary to MCAO, which will assist physicians in identifying appropriate patients earlier and offering personalized therapy.

## 2. Materials and Methods

### 2.1. Population and Design

From January 2018 to January 2025, a retrospective study was conducted, involving 101 NSCLC patients with MCAO who underwent therapeutic bronchoscopy. Inclusion criteria were as follows: (1) radiologically confirmed MCAO with ≥ 50% airway stenosis verified by bronchoscopy, (2) histologically confirmed NSCLC, and (3) completion of therapeutic bronchoscopy with complete clinical data. Exclusion criteria were as follows: (1) non‐MCAO‐related uncorrectable RF, (2) severe comorbidities precluding bronchoscopy (cardiac/hepatic/renal insufficiency or coagulopathy), and (3) loss to follow‐up within 3 months postprocedure.

**Figure 1:**
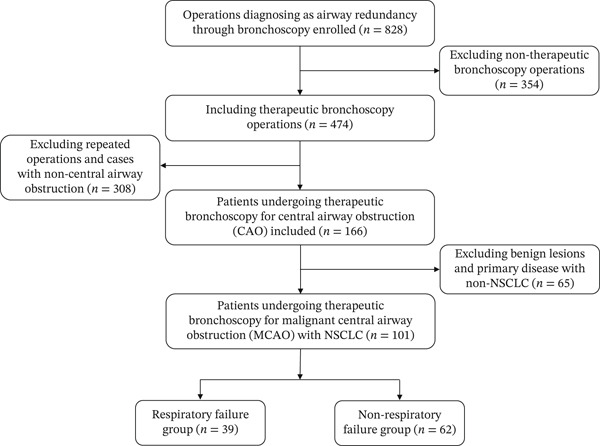
Study flowchart. NSCLC: non–small cell lung cancer.

According to whether the patient had MCAO‐related RF before bronchoscopy, patients were categorized into the RF group and the non‐RF group (Figure 1). The non‐RF group comprised patients with PaO_2_/FiO_2_ ≥ 300 mmHg. The RF group required (1) relevant clinical symptoms, (2) PaO_2_/FiO_2_ < 300 mmHg, and (3) exclusion of refractory hypoxemia arising from etiologies other than central airway obstruction, including but not limited to severe COPD, interstitial lung fibrosis, and massive pleural effusion.

### 2.2. Procedure

Before the procedure, all patients underwent (1) laboratory investigations (arterial blood gas, complete blood count [CBC], coagulation panel, and biochemistry profile), (2) ECOG performance status (PS) assessment, and (3) electrocardiography. MCAO location and severity were evaluated by chest computed tomography (CT). Postprocedural arterial blood gas analysis and chest CT were performed to assess treatment outcomes.

For patients with local anesthesia or general anesthesia, a flexible or rigid bronchoscope was chosen depending on the factual situation. Superimposed high‐frequency jet ventilation (Carl Reiner, Austria) was integrated with rigid bronchoscopy, with ventilation parameters calibrated based on the patient′s body weight [13]. Vital signs were closely monitored and recorded throughout the procedures, and the bispectral index (BIS) was recorded when necessary.

Mechanical debulking, thermal ablation, cryotherapy, and balloon dilatation were the commonly chosen methods in therapeutic bronchoscopy. Various sizes and specifications of self‐expandable metallic stents (partially covered or uncovered) and silicone stents could be placed if necessary. Individualize the selection and combination of these techniques according to type, location, severity, and other characteristics of airway obstruction [7, 14].

### 2.3. Measure and Definitions

This study established an electronic database using medical records from the electronic medical record system supplemented by telephone follow‐up data. Data extracted from the system encompassed demographics, clinical characteristics, sedation and procedural information, histopathology, laboratory and radiographic examinations, antitumor treatment, and survival over 2 years. The effectiveness of therapeutic bronchoscopy was analyzed in three timeframes: short‐term, intermediate‐term, and long‐term outcomes. To assess short‐term efficacy (≤ 1 week postprocedure), we compared changes in stenosis grade within patients and arterial blood gas parameters in the RF group. Intermediate‐term efficacy (3 months) was evaluated using changes in ECOG PS and MCAO‐related comorbidities. Long‐term outcomes were determined by 2‐year survival rates (Figure S1).

The degree of airway obstruction was determined by the Myer–Cotton grading method [15]. The MCAO‐related comorbidities recorded through chest imaging data include obstructive pneumonia or atelectasis and pleural effusion. The improvement of comorbidities was evaluated by comparing the chest imaging data before surgery and within 3 months after surgery. Technical success was evaluated using anatomical criteria and defined as reopening the airway lumen to > 50% of its normal diameter and linking it to an active region in the distal lung [16–18]. The operational complications were defined as any events related to therapeutic bronchoscopy within 24 h that required urgent reintervention, invasive resuscitation, or surgical intervention [19]. Complications included hemorrhage and hypoxemia. Hemorrhage was defined as intraprocedural blood loss > 100 mL that could not be controlled with argon plasma coagulation (APC) or diode laser. Hypoxemia was defined as SpO_2_ < 90% lasting for more than 1 min [20].

Overall survival (OS) refers to the duration from the first interventional procedure of the patient until death.

### 2.4. Statistical Analysis

An exploratory sample size calculation was conducted based on interim data from 16 patients to compare long‐term outcomes between RF and non‐RF groups. Assuming *α* = 0.05, a power of 90%, and an enrollment ratio of 1:1, the 1‐year mortality in the RF and non‐RF groups was 0.5 and 0.125, respectively, and a sample size of 27 patients in each group was calculated by PASS Version 2021.

Statistical analyses were performed using SPSS 26.0 and GraphPad Prism 9.5. Categorical variables are presented as frequency (percentage). Continuous variables are summarized as median (interquartile range). Statistical comparisons employed the Wilcoxon–Mann–Whitney test, chi‐square test, or Fisher′s exact test, as appropriate. Kaplan–Meier analysis estimated 2‐year survival rates. Univariate associations with OS were assessed using log‐rank tests. Variables with *p* values < 0.10 were entered into the Cox proportional hazards model for multivariate analysis. Statistical significance was set at a two‐sided *p* value < 0.05.

## 3. Results

### 3.1. Baseline Characteristics

The study cohort was predominantly male (86.1%) with a median age of 69.0 years. Most patients (89.1%) presented with advanced‐stage (III–IV) malignancy, predominantly squamous cell carcinoma (SCC; 75.2%). Upon admission, 58.4% of patients had ECOG PS 3–4; scores were significantly higher in the RF versus non‐RF group (*p* = 0.021). Twenty patients (19.8%) received no systemic antitumor therapy before or after therapeutic bronchoscopy due to compromised PS or chemotherapy refusal. Complete demographic and clinical characteristics are detailed in Table 1.

**Table 1:** Baseline characteristics.

Variables	Cohort			*p* value
	Total (*n* = 101)	RF (*n* = 39)	Non‐RF (*n* = 62)	
Age	69 (61.00–74.00)	69 (64.00–75.00)	69 (57.75–73.25)	0.025
Gender				0.810
Male	87 (86.1)	34 (87.2)	53 (85.5)	
Female	14 (13.9)	5 (12.8)	9 (14.5)	
Histopathology				0.948
Squamous cell carcinoma	76 (75.2)	30 (76.9)	46 (74.2)	
Adenocarcinoma	13 (12.9)	5 (12.8)	8 (12.9)	
Adenoid cystic carcinoma	4 (4.0)	1 (2.6)	3 (4.8)	
Others^a^	8 (7.9)	3 (7.7)	5 (8.1)	
Clinical stage				0.492
Stage II	11 (10.9)	5 (12.8)	6 (9.7)	
Stage III	60 (59.4)	25 (64.1)	35 (56.5)	
Stage IV	30 (29.7)	9 (23.1)	21 (33.9)	
ECOG PS				0.021
1–2	42 (41.6)	10 (25.6)	32 (51.6)	
3–4	59 (58.4)	29 (74.4)	30 (48.4)	
Systemic therapy				0.512
Positive	81 (80.2)	30 (76.9)	51 (82.3)	
Negative	20 (19.8)	9 (23.1)	11 (17.7)	

*Note:* Data are expressed in numbers (%) for dichotomic variables and in median values (range) for continuous variables.

Abbreviations: ECOG PS, Eastern Cooperative Oncology Group performance status; RF, respiratory failure.

^a^Others: adenosquamous carcinoma, sarcomatoid carcinoma, atypical carcinoid carcinoma, and chondrosarcoma.

Airway obstruction was classified as intraluminal in 44 patients (43.6%), extrinsic in 15 (14.9%), and mixed in 42 (41.6%). Severe airway obstruction (Myer–Cotton Grades III–IV) was observed in 94.1% of patients, with the left main bronchus being the most frequent site of stenosis (31.7%).

Compared with the non‐RF group, the most common obstruction location of patients in the RF group was the right main bronchus (*p* = 0.024). Details are shown in Table 2.

**Table 2:** Technical therapeutic bronchoscopy characteristics.

Variables		Cohort			*p* value
		Total (*n* = 101)	RF (*n* = 39)	Non‐RF (*n* = 62)	
Degree of airway stenosis^a^					0.323
Grade II		6 (5.9)	3 (7.7)	3 (4.8)	
Grade III		53 (52.5)	17 (43.6)	36 (58.1)	
Grade IV		42 (41.6)	19 (48.7)	23 (37.1)	
Site of lesion					0.024
Main trachea		15 (14.9)	8 (20.5)	7 (11.3)	
Left main bronchus		32 (31.7)	8 (20.5)	24 (38.7)	
Right main bronchus		28 (27.7)	16 (41.1)	12 (19.4)	
Right middle bronchus		26 (25.7)	7 (17.9)	19 (30.6)	
Types of obstruction present					0.326
Endoluminal		44 (43.6)	14 (35.9)	30 (48.4)	
Extrinsic		15 (14.9)	5 (12.8)	10 (16.2)	
Mixed		42 (41.6)	20 (51.3)	22 (35.5)	
Obstructive pneumonia					0.084
Yes		76 (75.2)	33 (84.6)	43 (69.4)	
Number of involved lung lobes					0.027
1		40 (39.6)	13 (33.3)	27 (43.5)	
2		25 (24.8)	12 (30.8)	13 (21.0)	
3		11 (10.9)	8 (20.5)	3 (4.8)	
Pleural effusion					0.013
Yes		32 (31.7)	18 (46.2)	14 (22.6)	
Anesthesia					0.257
Moderate sedation		46 (45.5)	15 (38.5)	31 (50.0)	
General anesthesia		55 (54.5)	24 (61.5)	31 (50.0)	
Type of bronchoscopy					0.176
Flexible		50 (49.5)	16 (41.0)	34 (54.8)	
Rigid		51 (50.5)	23 (59.0)	28 (45.2)	
Interventional techniques					
Cryotherapy		77 (76.2)	31 (79.5)	46 (74.2)	0.543
Thermal ablation		55 (54.5)	22 (56.4)	33 (53.2)	0.754
Mechanical debulking		74 (73.3)	28 (71.8)	46 (74.2)	0.791
Stent		9 (8.9)	3 (7.7)	6 (9.7)	1.000
Integrated therapy^b^		80 (79.2)	29 (74.4)	51 (82.3)	0.341
Followed complications					0.147
No		99 (98.0)	37 (94.9)	62 (100.0)	
Hemorrhage		1 (1.0)	1 (2.6)	0 (0.0)	
Hemorrhage with hypoxia		1 (1.0)	1 (2.6)	0 (0.0)	
Result					0.521
Success		94 (93.1)	35 (89.7)	59 (95.2)	
Failure		7 (6.9)	4 (10.3)	3 (4.8)	

*Note:* Data are expressed in numbers (%).

^a^The degree of airway stenosis was determined by the Myer–Cotton grading method which is based on the percentage of reduction in cross‐sectional area. Grade I, ≤ 50% luminal stenosis; Grade II, 51%–70% luminal stenosis; Grade III, 71%–99% luminal stenosis; Grade IV, no lumen.

^b^Integrated therapy: a combination of these techniques.

### 3.2. Safety and Efficacy of Bronchoscopy

Among 101 operations, 94 (93.1%) achieved technical success. The remaining seven cases constituted technical failures, defined as achieving a reopened airway lumen < 50% of normal diameter due to safety concerns. Acute complications occurred in two patients (2.0%): one case of intraoperative bleeding requiring emergency intervention and one case of pneumothorax leading to cardiorespiratory arrest. Both patients received tracheal intubation, were transferred to the ICU, and were subsequently discharged successfully. No procedure‐related mortality occurred (Table 2).

**Figure 2:**
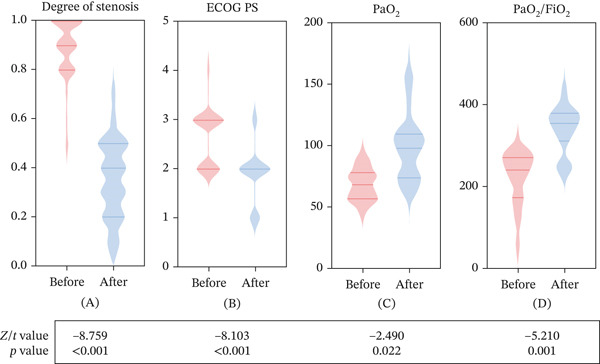
Short‐term and intermediate‐term efficacy. (A, B) A significant difference in the degree of airway stenosis and the ECOG PS score before and after therapy (*n* = 101). (C, D) A significant difference in the PaO_2_ and the PaO_2_/FiO_2_ before and after therapy among the RF group (*n* = 39).

**Figure 3:**
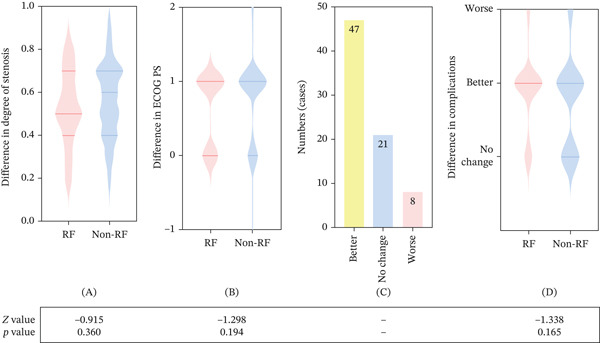
Comparative analysis. (A, B) No significant difference between the RF group and the non‐RF group in the improvement of the airway stenosis (*p* = 0.360) and the ECOG PS (*p* = 0.194). (C) Difference in comorbidities before and after therapy. (D) No significant difference between the RF group and the non‐RF group in the improvement of MCAO‐related comorbidities (*p* = 0.165). ECOG PS: Eastern Cooperative Oncology Group performance status; RF: respiratory failure; MCAO: malignant central airway obstruction.

Following interventional bronchoscopy, both airway obstruction severity and ECOG PS showed significant improvement (*p* < 0.001 for each) (Figure 2A,B). The RF group patients (*n* = 39) also exhibited notable improvement in both PaO_2_ (*p* = 0.022) and the oxygenation index (*p* = 0.001) (Figure 2C,D). Among patients with MCAO‐related comorbidities (*n* = 76), therapeutic bronchoscopy yielded clinical improvement in 61.8% of cases (Figure 3C). In patients with poor baseline status precluding systemic antitumor therapy (*n* = 36), 80.56% achieved general improvement and began to receive adjuvant treatment within 30 days (the median time was 7 days) (Figure S2). There was no difference in the technical success (*p* = 0.521), complications (*p* = 0.147), short‐term efficacy (*p* = 0.360 for the degree of stenosis), and intermediate‐term efficacy (*p* = 0.194 for ECOG PS and *p* = 0.165 for improvement of comorbidities) between the RF group and the non‐RF group (Figure 3A,B,D).

### 3.3. Survival Outcomes

Over the median follow‐up duration of 20.0 months (3.1–24.0 months), 51 cases (50.50%) resulted in mortality, 6 cases (5.94%) were lost to follow‐up, and 44 cases (43.56%) remained alive. Four patients (4.4%) died from progression of primary disease within 30 days after operation, including one with SCC involving the esophagus, who died owing to hematemesis on the 4th day after esophageal stent implantation.

**Figure 4:**
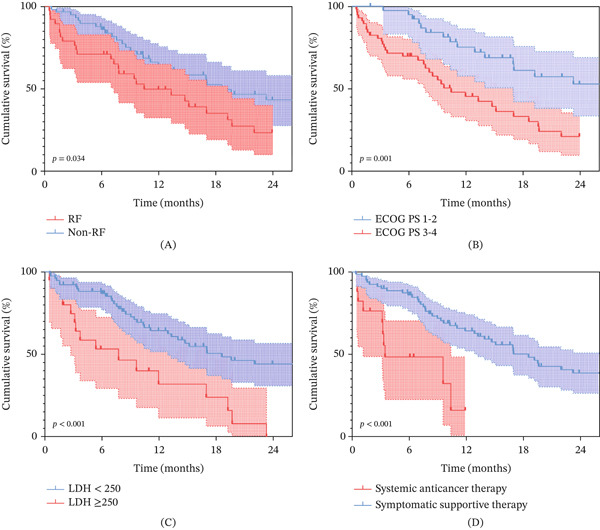
Survival analysis. Kaplan–Meier curve for 2‐year survival based on (A) the RF grouping (log − rank *χ* ^2^ = 4.494, *p* = 0.034), (B) the ECOG PS grouping (log − rank *χ* ^2^ = 10.795, *p* = 0.001), (C) the serum LDH grouping (log − rank *χ* ^2^ = 14.081, *p* < 0.001), and (D) the systemic anticancer therapy grouping (log − rank *χ* ^2^ = 14.893, *p* < 0.001). RF: respiratory failure; ECOG PS: Eastern Cooperative Oncology Group performance status; LDH: lactate dehydrogenase.

Given that adenoid cystic carcinoma (ACC) is classified as a tumor with low malignancy, four patients with ACC whose survival periods were more than 2 years were excluded from the survival analysis. For the 97 NSCLC patients, the median OS was 16.98 months, and the survival rates at 1, 3, 6, 9, 12, and 18 months were 94.8%, 87.6%, 80.0%, 67.4%, 58.1%, and 43.7%, respectively. Log‐rank univariate analysis revealed statistically significant differences in OS between the following groups: the RF group and the non‐RF group (10.46 vs. 18.62 months; log − rank *χ* ^2^ = 4.494, *p* = 0.034) (Figure 4A), the ECOG PS 3–4 group and the ECOG PS 1–2 group (10.46 vs. 26.89 months; log − rank *χ* ^2^ = 10.795, *p* = 0.001) (Figure 4B), the lactate dehydrogenase (LDH) ≥ 250 U/L group and the LDH < 250 U/L group (7.77 vs. 18.62 months; log − rank *χ* ^2^ = 14.081, *p* < 0.001) (Figure 4C), and the symptomatic supportive therapy group and the systematic antitumor therapy group (3.54 vs. 18.62 months; log − rank *χ* ^2^ = 14.893, *p* < 0.001) (Figure 4D).

**Figure 5:**
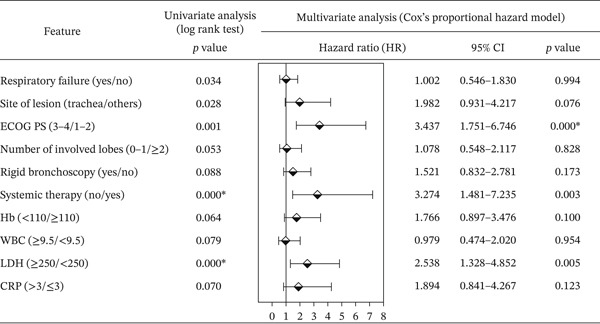
Univariate and multivariate analysis. CI: confidence interval; Hb: hemoglobin; WBC: white blood cell count; LDH: lactate dehydrogenase; CRP: C‐reactive protein. ∗ *p* value < 0.001.

Select factors related to survival by univariate analysis (*p* < 0.1) are assigned in Table S1, and a variable Cox risk regression model was employed for further analysis. Cox proportional hazards regression showed that the ECOG PS of 3–4 points (HR = 3.437, 95% CI: 1.751–6.746, *p* < 0.001), serum LDH ≥ 250 U/L (HR = 2.538, 95% CI: 1.328–4.852, *p* = 0.005), and lack of systemic antitumor therapy regularly (HR = 3.274, 95% CI: 1.481–7.235, *p* = 0.003) were independent risk factors affecting the 2‐year survival of NSCLC‐related MCAO patients after therapeutic bronchoscopy. Notably, the presence of correctable RF related to MCAO was not correlated with OS (HR = 1.002, 95% CI: 0.546–1.830, *p* = 0.994) (Figure 5).

## 4. Discussion

**Figure 6:**
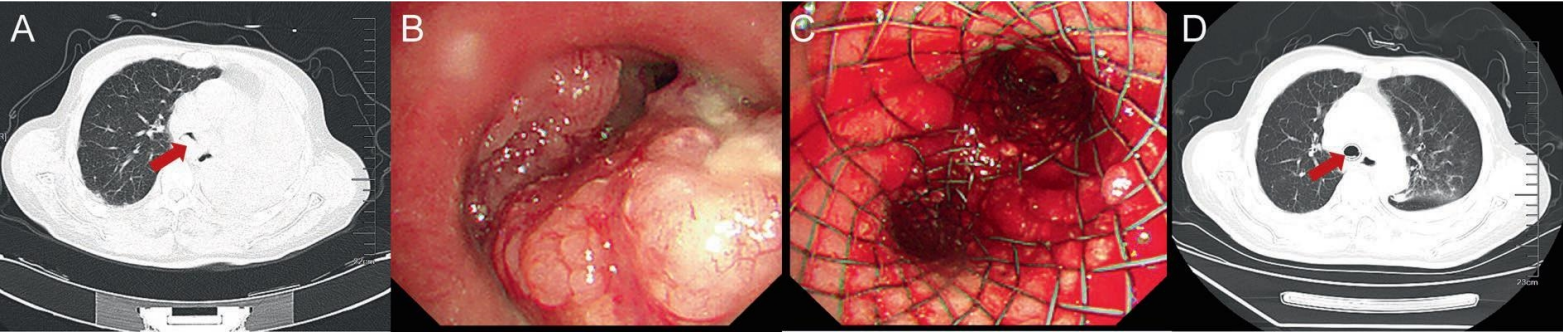
The improvement of MCAO‐related comorbidities after the therapeutic bronchoscopy. (A) Chest computed tomography (CT) revealed the right‐sided obstructive pneumonia and atelectasis secondary to stenosis of the right main bronchus (red arrow). (B) Bronchoscopy revealed a protruding mass in the right main bronchus. (C) The airway lumen was fully opened after the placement of an airway metal stent. (D) CT showed a marked improvement of obstructive pneumonia and atelectasis after metal stent placement (red arrow).

Epidemiological studies indicate that approximately one‐third of NSCLC patients develop MCAO at initial diagnosis or during disease progression. Primary symptoms include cough, dyspnea, hemoptysis, and related manifestations [7]. With advances in respiratory interventional techniques, therapeutic bronchoscopy centered on endoluminal therapy has become a cornerstone of multidisciplinary management for MCAO patients. Therapeutic bronchoscopy achieved high clinical success rates in NSCLC‐related MCAO, demonstrating significant improvements in ventilation, control of comorbidities, and restoration of lung function. As shown in Figure 6, a representative patient presented with carinal stenosis and complete left lung collapse complicated by RF. This critical condition precluded systemic antitumor therapy. Conventional management would have been limited to palliative care. Following Y‐stent placement via therapeutic bronchoscopy, near‐normal airway anatomy and complete left lung re‐expansion were achieved. Concomitant clinical improvement enabled subsequent chemotherapy and immunotherapy. This demonstrates that localized airway intervention facilitates systemic therapy through physiological optimization, ultimately extending survival in advanced lung cancer.

Whereas therapeutic bronchoscopy demonstrates favorable technical success and safety profiles, standardized criteria for defining technical success remain lacking. Current literature [17–19, 21, 22] typically defines technical success as achieving ≥ 50% restoration of airway lumen cross‐sectional area with re‐established anatomical continuity to functional distal lung tissue following bronchoscopic intervention. Applying this definition, our study achieved a 93.1% technical success rate. Median airway stenosis decreased from 90% preprocedure to 40% postintervention, aligning with outcomes reported in prior studies [17, 18, 23, 24].

As an invasive procedure, interventional bronchoscopy carries the risk of various complications. In reference to previous researches, the incidence of complications was rare, ranging from 3.9% to 15.6% [18, 19, 25, 26], with the mortality ranging from 0% to 1.3% [18, 27, 28]. Preoperative ASA scores > 3 and emergency or repeated procedures appear associated with increased risk of intraoperative complications [19]. In our cohort, only two patients (2.0%) required clinical intervention for complications during or within 24 h postprocedure. Both recovered fully with intraoperative rescue and intensive care. No procedure‐related mortality occurred. The 30‐day postprocedural mortality was 4.12%, demonstrating greater safety than literature benchmarks, likely attributable to stringent patient selection and limited sample size. The small complication cohort precluded statistically meaningful risk factor analysis.

The role of therapeutic bronchoscopy in managing RF remains debated. The conventional view regards severe RF as a relative contraindication to bronchoscopy [29], primarily for two reasons: First, reduced anesthesia tolerance substantially increases procedural risk; second, airway manipulation may exacerbate RF and precipitate critical events such as hypoxemic cardiac arrest. Although some studies report benefits in selected patients, current evidence stems predominantly from small studies (typically < 10 cases per cohort), precluding rigorous efficacy evaluation [12, 24]. We specifically analyzed the MCAO‐induced RF subgroup, excluding cases secondary to chronic pulmonary pathologies (e.g., severe COPD or interstitial fibrosis) or acute conditions like ARDS. This subgroup demonstrated significant postprocedural improvements in PaO_2_ (*p* = 0.022) and oxygenation index (*p* = 0.001) versus baseline, indicating that MCAO‐induced RF is potentially reversible with endoluminal therapy. Crucially, technical success (89.7% vs. 95.2%; *p* = 0.521) and safety outcomes (complication rates: 2.6% vs. 0.0%; *p* = 0.147) were comparable to non‐RF MCAO patients. These results offer novel evidence to inform management of such acute presentations.

Despite significantly worse baseline ECOG PS in RF group patients versus non‐RF controls, 64.1% (25/39) achieved clinically significant improvement (≥ 1 grade reduction) in ECOG PS status within 3 months after technically successful bronchoscopy. This functional improvement enabled subsequent systemic anticancer therapy. Multivariate analysis showed comparable 2‐year survival (adjusted HR = 1.01, 95% CI: 0.48–2.13; *p* = 0.994). These findings indicate that for reversible RF secondary to airway obstruction, therapeutic bronchoscopy should be considered promptly, even in patients with poor baseline PS, to enhance functional status and potentially improve survival outcomes.

Multivariate Cox regression identified three independent predictors of survival after bronchoscopy in NSCLC‐associated MCAO patients. Firstly, ECOG PS 3–4 (HR = 3.44, 95% CI: 2.16–5.48; *p* < 0.001) was the primary risk factor with dual clinical significance. This status indicates severely compromised functional reserve, reducing procedural tolerance as evidenced by established associations with complication risk [19] and technical failure [23, 30]. Simultaneously, it reflects diminished physiological reserve that independently correlates with poor outcomes [9, 18]. Secondly, absence of systemic anticancer therapy (HR = 3.27, 95% CI: 1.51–7.09; *p* = 0.003) was a critical predictor. This aligns with therapeutic consensus supporting combined local–systemic therapy for NSCLC patients with acute complications [31–33]. Although bronchoscopy alleviates mechanical obstruction, it cannot alter tumor biology. Thus, omitting systemic therapy increases the risks of local recurrence and distant metastasis. Thirdly, serum LDH ≥ 250 U/L independently predicted poor prognosis (HR = 2.54, 95% CI: 1.32–4.88; *p* = 0.005). LDH facilitates tumor progression through the Warburg effect, wherein malignant cells preferentially utilize glycolysis. This metabolic shift accumulates lactate, acidifying the microenvironment and promoting invasion/metastasis. Consequently, serum LDH reflects tumor metabolic burden [34] and serves as an independent prognostic indicator for NSCLC [35]. Our findings confirm LDH′s prognostic utility specifically in MCAO patients, extending both the multicenter observations by Nagano et al. and the MCAO‐specific analyses by Xing et al. [9, 36]. Persistent postprocedural LDH elevation warrants therapeutic reassessment, which may include regimen intensification or palliative interventions.

Certain limitations in this article were as follows. First, the single‐center design and limited sample size may have introduced bias. Second, ethical considerations precluded establishing a nonintervention control group for patients requiring bronchoscopy, which limited our ability to compare the effect of endobronchial therapy on survival in MCAO patients. Furthermore, as most RF patients required oxygen supplementation to tolerate examinations, we used PaO_2_/FiO_2_ as the grouping criterion. This approach may have misclassified some RF cases. Third, this retrospective study limits the inclusion of additional follow‐up time points and relevant scoring indicators, thereby restricting a more comprehensive assessment of the impact of endobronchial therapy on patients′ quality of life.

## 5. Conclusion

In conclusion, therapeutic bronchoscopy demonstrates both safety and efficacy in managing NSCLC‐associated MCAO and should be considered when RF is secondary to airway narrowing. Poor ECOG PS, higher serum LDH, and absence of systemic antitumor therapy are independent risk factors for reduced survival time in MCAO patients.

## Funding

No funding was received for this manuscript.

## Conflicts of Interest

The authors declare no conflicts of interest.

## Supporting information


**Supporting Information** Additional supporting information can be found online in the Supporting Information section. Supporting Information. Table S1: Correlation of clinical factors and 2‐year survival (log‐rank univariate analysis). ^a^Others: adenoid cystic carcinoma, sarcomatoid carcinoma, atypical carcinoid carcinoma, and chondrosarcoma. ^b^Others: main bronchus and right middle bronchus. ^c^Determined by the Myer–Cotton grading method. ^d^Eastern Cooperative Oncology Group performance status. ^e^Hb: hemoglobin. ^f^WBC: white blood cell count. ^g^LDH: lactate dehydrogenase. ^h^ALB: albumin. ^I^CRP: C‐reactive protein. Figure S1: Evaluating the effectiveness of therapeutic bronchoscopy. RF: respiratory failure; ECOG PS: Eastern Cooperative Oncology Group performance status; MCAO: malignant central airway obstruction; ACC: adenoid cystic carcinoma. Figure S2: Interval from therapeutic bronchoscopies to systemic antitumor treatments in patients who hardly tolerate adjuvant treatment before operations (*n* = 29).

## Data Availability

The data that support the findings of this study are available on request from the corresponding author. The data are not publicly available due to privacy or ethical restrictions.
