# Supplementary material for: Outcomes and Prognostic Analysis of Therapeutic Bronchoscopy in Non–Small Cell Lung Cancer (NSCLC) Patients With Respiratory Failure Secondary to Malignant Central Airway Obstruction (MCAO)
Source: Pulm Med. 2026 Jul 20;2026:4702786. doi: 10.1155/pm/4702786 (PMC13382534; doi:10.1155/pm/4702786)
Supplement: Supplementary file 1 — Supporting Information Additional supporting information can be found online in the Supporting Information section. Supporting Information. Table S1: Correlation of clinical factors and 2‐year survival (log‐rank univariate analysis). aOthers: adenoid cystic carcinoma, sarcomatoid carcinoma, atypical carcinoid carcinoma, and chondrosarcoma. bOthers: main bronchus and right middle bronchus. cDetermined by the Myer–Cotton grading method. dEastern Cooperative Oncology Group performance status. eHb: hemoglobin. fWBC: white blood cell count. gLDH: lactate dehydrogenase. hALB: albumin. ICRP: C‐reactive protein. Figure S1: Evaluating the effectiveness of therapeutic bronchoscopy. RF: respiratory failure; ECOG PS: Eastern Cooperative Oncology Group performance status; MCAO: malignant central airway obstruction; ACC: adenoid cystic carcinoma. Figure S2: Interval from therapeutic bronchoscopies to systemic antitumor treatments in patients who hardly tolerate adjuvant treatment before operations (n = 29). [file PM-2026-4702786-s001.doc]

**Supplementary Table**

**Table S1** Correlation of clinical factors and 2-year survival (Log-rank univariate analysis)

| Variables | Median survival time (months） | Log-rank χ^2^  value | P value |
| --- | --- | --- | --- |
| Respiratory failure  Positive  Negative | 10.46  18.62 | 4.494 | **0.034** |
| Histopathology  Squamous cell carcinoma  Adenocarcinoma  Others ^a^ | 17.02  7.28  11.98 | 2.975 | 0.226 |
| Site of lesion  Main trachea  Others^b^ | 8.16  17.05 | 4.855 | **0.028** |
| Degree of airway stenosis^c^  Grade II  Grade III-IV | 6.39  17.02 | 2.576 | 0.109 |
| Clinical stage  Stage Ⅱ  Stage Ⅲ  Stage Ⅳ | 8.52  14.00  18.62 | 0.757 | 0.685 |
| ECOG PS score^d^  1-2 points  3-4 points | 26.89  10.46 | 10.795 | **0.001** |
| Initial diagnosis  Yes  No | 18.62  14.75 | 1.205 | 0.272 |
| Related comorbidities  Yes  No | 14.00  22.03 | 0.704 | 0.401 |
| Number of involved lung lobes  0-1  ≥2 | 19.61  9.64 | 3.747 | **0.053** |
| Pleural effusion  Yes  No | 11.97  17.02 | 0.495 | 0.482 |
| Anesthesia  Moderate sedation  General anesthesia | 17.02  14.00 | 0.879 | 0.348 |
| Type of bronchoscopy  Flexible  Rigid | 17.05  14.00 | 2.906 | **0.088** |
| Followed complications  Yes  No | 0.56  17.02 | 2.461 | 0.117 |
| Technical success  Yes  No | 15.18  18.62 | 0.141 | 0.707 |
| Systematic anti-tumor therapy  Yes  No | 18.62  3.54 | 14.893 | **＜0.001** |
| Hb^e^  <110 g/L  ≥110 g/L | 11.97  18.62 | 3.423 | **0.064** |
| WBC^f^  <9.5 *10^9^/L  ≥9.5 *10^9^/L | 18.62  9.05 | 3.081 | **0.079** |
| LDH^g^  <250 U/L  ≥250 U/L | 18.62  7.77 | 14.081 | **＜0.001** |
| ALB^h^  <40 g/L  ≥40 g/L | 11.11  19.25 | 2.075 | 0.150 |
| CRP^i^  ≤3 mg/L  >3 mg/L | 22.03  12.95 | 3.290 | **0.070** |

^a^ Others: adenosquamous carcinoma, sarcomatoid carcinoma, atypical carcinoid carcinoma, chondrosarcoma; ^b^ Others: main bronchus and right middle bronchus; ^c^ Determined by the Myer-Cotton grading method; ^d^ Eastern Cooperative Oncology Group Performance Status; ^e^ Hb: Hemoglobin; ^f^ WBC: white blood cell count; ^g^ LDH: Lactate dehydrogenase; ^h^ ALB: Albumin; ^i^ CRP: C-reactive protein.

**Supplementary Figure**

**
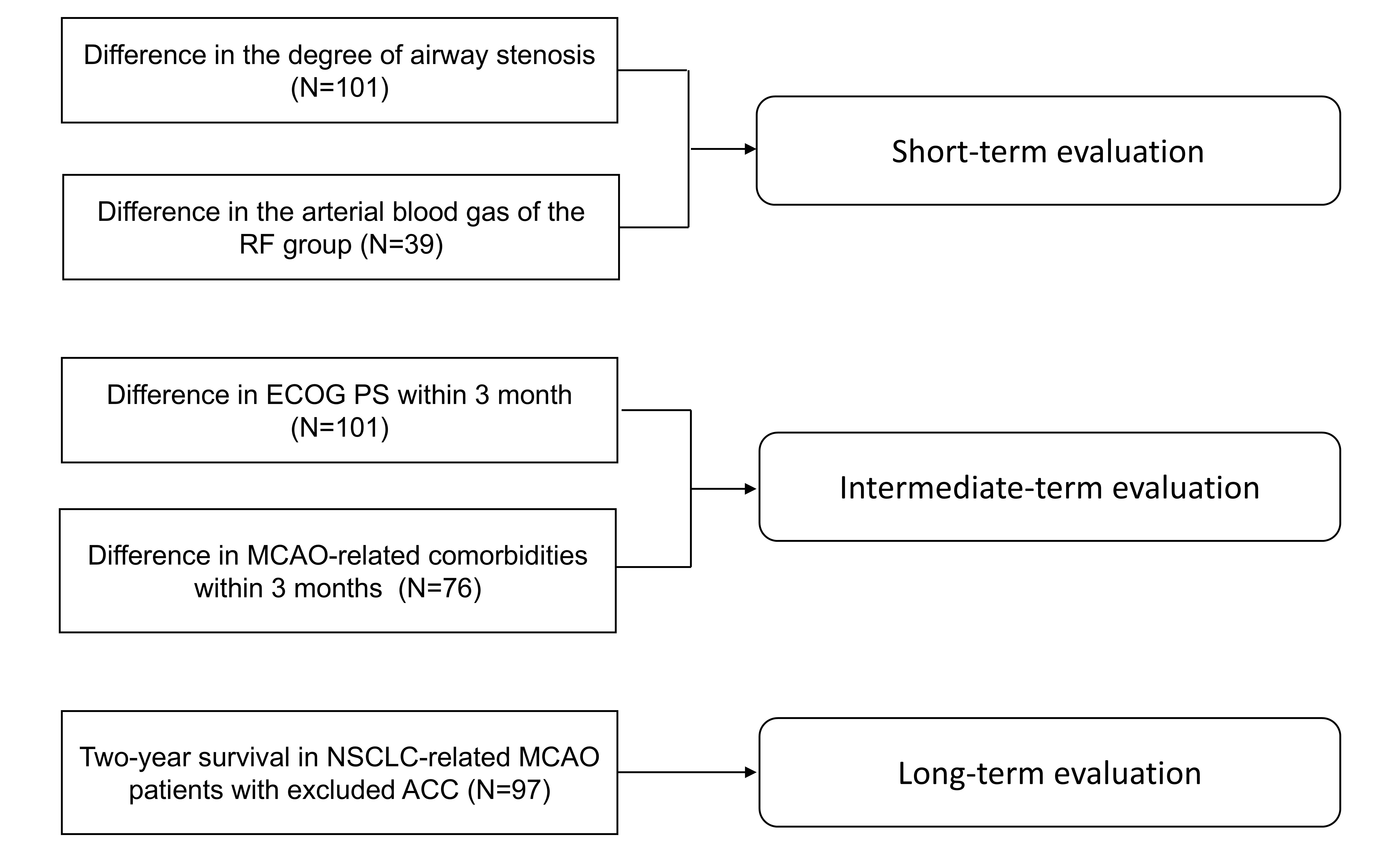
**

**Figure S1** Evaluating the effectiveness of therapeutic bronchoscopy

RF: respiratory failure; ECOG PS: Eastern Cooperative Oncology Group performance status; MCAO: malignant central airway obstruction; ACC: adenosquamous cell carcinoma


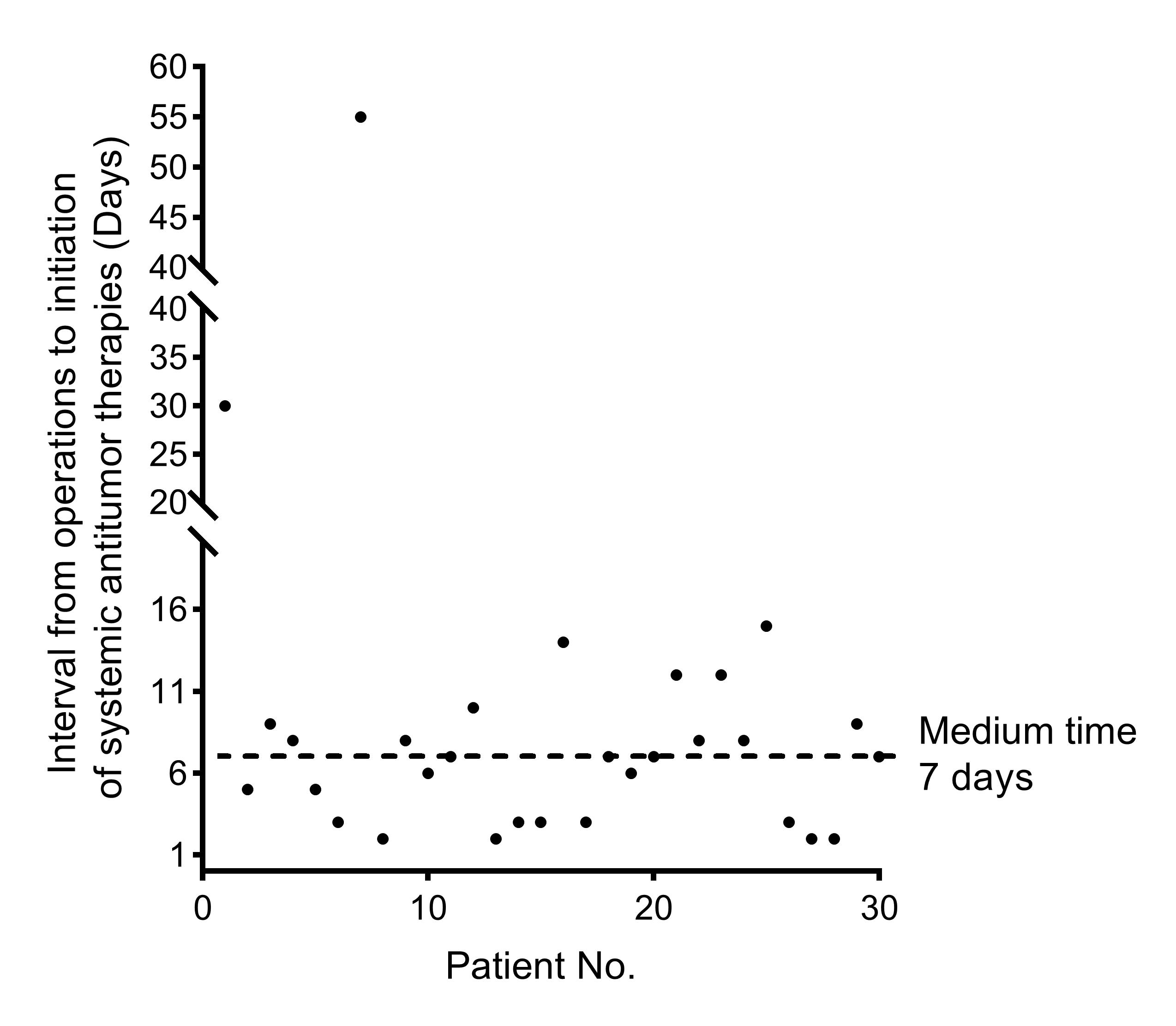


**Figure S2** Interval from therapeutic bronchoscopies to systemic anti-tumor treatments in patients who hardly tolerate adjuvant treatment before operations(n=29)
